# Spin excitations near the pressure-induced antiferromagnetic transition in SrCu_2_(BO_3_)_2_

**DOI:** 10.1107/S1600576726003705

**Published:** 2026-05-14

**Authors:** Jeppe Jon Cederholm, Andrea Piovano, Alexandre Ivanov, Stefan Klotz, Arno Hiess, Ursula B. Hansen, Ekaterina Pomjakushina, Mohamed E. Zayed, Ellen Fogh, Henrik M. Rønnow

**Affiliations:** a Institut Laue–Langevin 71 avenue des Martyrs CS 20156 38042 Grenoble Cedex 9 France; b Laboratory for Quantum Magnetism, Institute of Physics, École Polytechnique Fédérale de Lausanne (EPFL), CH-1015 Lausanne, Switzerland; c Sorbonne Université, UMR CNRS 7590, Institut de Minéralogie, de Physique des Matériaux et de Cosmochimie (IMPMC), 4 Place Jussieu, 75005 Paris, France; d European Spallation Source ERIC PO Box 176 22100 Lund Sweden; e Center for Neutron and Muon Sciences, Paul Scherrer Institute, CH-5232 Villigen PSI, Switzerland; f Department of Physics, Carnegie Mellon University in Qatar, Education City, PO Box 24866, Doha, Qatar; g Physics Department, Technical University of Munich, D-85748 Garching, Germany; h Center for Quantum Engineering (ZQE), Technical University of Munich, D-85748 Garching, Germany; European Spallation Source ERIC, Sweden

**Keywords:** frustrated magnetism, high-pressure studies, inelastic neutron scattering, quantum many-body systems, antiferromagnets

## Abstract

SrCu_2_(BO_3_)_2_ is a frustrated quantum magnet which realizes the Shastry–Sutherland model. The system exhibits a sequence of pressure-induced phases, including a long-range antiferromagnetically ordered state. We have performed inelastic neutron scattering experiments on a single crystal at 4.2 (2) GPa, in the antiferromagnetic phase. The observed dispersive spin excitations were modeled using linear-spin-wave theory to extract exchange parameters. Our results are consistent with previously reported parameters at higher pressures and confirm the existence of a Goldstone mode as well as underlining the importance of the interlayer coupling. These results complement previous high-pressure studies and provide further input for theoretical models of SrCu_2_(BO_3_)_2_ under extreme conditions.

## Abstract 2

Inelastic neutron scattering on SrCu_2_(BO_3_)_2_ at 4.2 GPa reveals dispersive spin excitations in the antiferromagnetic phase, consistent with linear-spin-wave theory and highlighting the key role of interlayer coupling.

## 1. Introduction

Frustrated quantum magnets are a fertile ground for discovering exotic states of matter and unconventional excitations. In this context, the Shastry–Sutherland model (SSM) describes a two-dimensional network of orthogonal spin-1/2 dimers coupled by intra- and interdimer exchange interactions, respectively, *J* and *J*′ (Sriram Shastry & Sutherland, 1981 ▸). For small *J*′/*J*, the ground state is an exact product of singlet dimers, whereas increasing *J*′/*J* introduces frustration and drives the system through a sequence of magnetically correlated phases, including a plaquette state and ultimately long-range antiferromagnetic (AFM) order (Corboz & Mila, 2013 ▸; Boos *et al.*, 2019 ▸). The model also hosts magnetization-plateau phases in applied magnetic fields (Momoi & Totsuka, 2000 ▸; Corboz & Mila, 2014 ▸).

**Figure 1:**
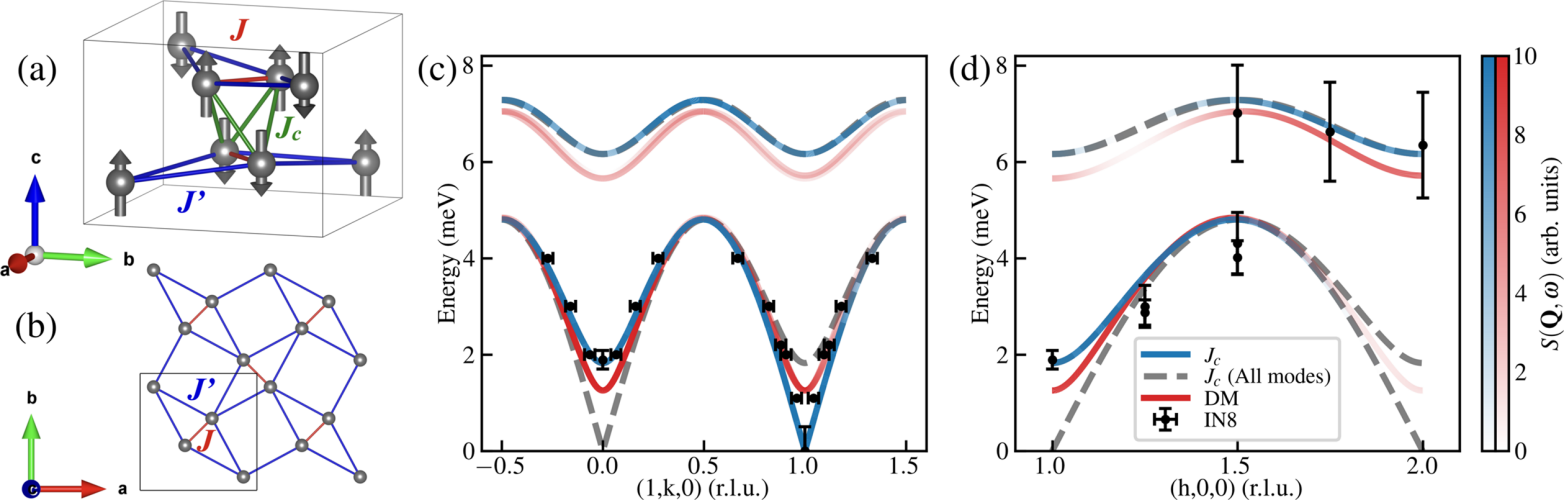
(*a*) Unit cell of SrCu_2_(BO_3_)_2_ showing only the magnetic Cu^2+^ ions and the accompanying AFM structure. (*b*) A single layer of Cu^2+^ ions corresponding to the Shastry–Sutherland lattice. (*c*, *d*) Comparison between predicted and measured excitation spectra for the AFM phase of SrCu_2_(BO_3_)_2_ at *T* = 4.6 (1) K and *P* = 4.2 (2) GPa. The blue and gray dashed curves include the interlayer interaction *J*_c_; the gray curve shows all calculated modes, while the blue curve shows the corresponding intensity. This model yields both a gapped and a gapless Goldstone mode at the zone center, with intensity appearing at (1, 0, 0) and (1, 1, 0), respectively. In contrast, the red curves correspond to a model with DM interactions and no interlayer coupling, as detailed by Fogh *et al.* (2024 ▸), resulting in a single gapped mode at both positions.

The compound SrCu_2_(BO_3_)_2_ provides an almost ideal realization of the SSM (Miyahara & Ueda, 1999 ▸; Kageyama *et al.*, 1999*b* ▸). It is characterized by interactions *J* and *J*′, respectively, inside and between the dimers, as illustrated in Figs. 1 ▸(*a*) and 1 ▸(*b*). At ambient pressure, SrCu_2_(BO_3_)_2_ is well characterized by the SSM with *J*′/*J* = 0.63 (Miyahara & Ueda, 2000 ▸; Shi *et al.*, 2022 ▸), placing it near the boundary between the singlet-dimer and plaquette regimes. Experiments have confirmed the singlet-dimer ground state (Miyahara & Ueda, 1999 ▸; Kageyama *et al.*, 2000 ▸; Gaulin *et al.*, 2004 ▸; Kakurai *et al.*, 2005 ▸) and shown that applying hydrostatic pressure increases *J*′/*J*, sequentially inducing a plaquette phase (Zayed *et al.*, 2017 ▸; Jiménez *et al.*, 2021 ▸; Waki *et al.*, 2007 ▸; Sakurai *et al.*, 2018 ▸) and eventually long-range AFM order (Haravifard *et al.*, 2014 ▸; Guo *et al.*, 2020 ▸). Recently an additional quantum-spin-liquid (QSL) phase has been predicted to reside between the plaquette and AFM phases, but whether this is also realized in SrCu_2_(BO_3_)_2_ remains an open question (Corboz *et al.*, 2026 ▸). However the close correspondence between model and material makes SrCu_2_(BO_3_)_2_ an ideal platform for testing theoretical predictions for the highly frustrated SSM.

Going beyond 4 GPa, the system undergoes a change in crystal symmetry from tetragonal to monoclinic. Below the monoclinic distortion there have been reports of indirect AFM signatures at low temperature (Guo *et al.*, 2020 ▸). Above the monoclinic distortion there is strong AFM order, setting in at a high temperature of about 120 K (Zayed, 2010 ▸; Haravifard *et al.*, 2014 ▸). Recently our own inelastic neutron scattering experiment enabled quantitative determination of the mag­netic interaction parameters in SrCu_2_(BO_3_)_2_ at 5.5 GPa. The spin waves can be described by the same *J*′ and *J* in the plane but require a dramatic increase in *J*′/*J* and the addition of an interlayer coupling *J*_c_ (Fogh *et al.*, 2024 ▸). In the model, the orthogonal arrangement of the dimers together with the AFM spin arrangement [Fig. 1 ▸(*a*)] gives rise to a change in mode intensity such that at (1, 0, 0) one observes a gapped mode whereas at (1, 1, 0) the spectral weight is found in the Goldstone mode [Figs. 1 ▸(*c*) and 1(*d*)]. The splitting between the modes is then given by the interlayer coupling. However, in the previous study, the spectrum at (1, 1, 0) was not directly probed and, although unlikely, a model including a Dzyaloshinskii–Moriya (DM) interaction could also explain the observations. Therefore, to pin down the origin of the observed gap at (1, 0, 0) one needs to study the spectrum at (1, 1, 0).

Here we present complementary inelastic neutron scattering measurements performed at pressures close to the phase boundary between the tetragonal and monoclinic symmetries. We determine the evolution of the spin excitation spectrum and extract exchange interaction parameters from fits within linear-spin-wave theory. We directly confirm the existence of a Goldstone mode and thereby underline the importance of the interlayer interaction in SrCu_2_(BO_3_)_2_, providing further constraints on theoretical descriptions of its behavior under pressure.

## 2. Experimental details

A single crystal of SrCu_2_(BO_3_)_2_ grown by the floating-zone method (Kageyama *et al.*, 1999*a* ▸; Jorge *et al.*, 2004 ▸) was cut into a disc with a diameter of 4 mm, a thickness of 1.3 mm and a total weight of 36 mg. The crystal was oriented with **Q** = (*q*_*h*_, *q*_*k*_, 0) in the horizontal scattering plane. The sample was mounted in a Paris–Edinburgh press using a TiZr gasket assembly, Zr-toughened alumina anvils and deuterated 4:1 methanol–ethanol as pressure medium following the procedure detailed by Fogh *et al.* (2024 ▸). However, two improvements were implemented: (1) Cooling of the top gasket using liquid nitrogen to freeze the pressure transmitting fluid and thereby avoid the trapping of air in the sample chamber when it is placed on the lower gasket. (2) Inclusion of a 36 mg ring-shaped piece of Pb placed around the sample for pressure determination. The nuclear 111 Bragg peak of Pb was used to calibrate the pressure by using the well characterized equation of state (Strässle *et al.*, 2014 ▸). The final pressure at 4.6 K was 4.2 (2) GPa with a pressure drop of less than 10% upon cooling from room temperature. Tracking the 400 reflection during pressure increase revealed a small shift from *h* = 4.000 to *h* = 4.025 reciprocal lattice units (r.l.u.), corresponding to an in-plane lattice contraction of approximately 0.6% (Δ*a* ≈ −0.056 Å for *a*_0_ = 8.995 Å), consistent with previous studies (Loa *et al.*, 2005 ▸). Note that we stay with the tetragonal notation throughout the experiment, which is a good approximation in this geometry with the *a* and *b* lattice parameters changing less than 1% at our maximum pressure and with a monoclinic angle of β = 94° (Loa *et al.*, 2005 ▸; Haravifard *et al.*, 2014 ▸). Hence, the monoclinic distortion mainly results in a sliding of the Cu^2+^ layers with respect to each other out of plane, but the symmetry within the layers stays approximately tetragonal.

On the thermal triple-axis neutron spectrometer, IN8 (Piovano & Ivanov, 2023 ▸), at the Institute Laue–Langevin, we worked with a pyrolytic graphite [PG(200)] analyzer and Si(111) monochromator, both double focused. A PG filter was placed between sample and analyzer to suppress higher-order scattering. Two different final wavenumbers were used during the experiment, *k*_f_ = 2.662 Å^−1^ and *k*_f_ = 1.97 Å^−1^, giving an elastic line resolution full width at half-maximum of, respectively, 1.22 and 0.51 meV. Within each measurement the final wavevector was kept constant but the incident wavevector was changed to realize adequate energy transfer. A series of constant-*Q* and constant-*E* scans were performed. In addition, we followed the temperature evolution of the magnetic Bragg peak 100 upon heating (Zayed *et al.*, 2025 ▸).

An additional IN8 measurement was performed at 3.6 GPa using a different load of the Paris–Edinburgh cell under otherwise identical instrumental conditions. A 60 mg sample cut from the same crystal growth was used, corresponding to a pressure just below the transition to the monoclinic phase (Fogh *et al.*, 2021 ▸).

## 3. Results and discussion

**Figure 2:**
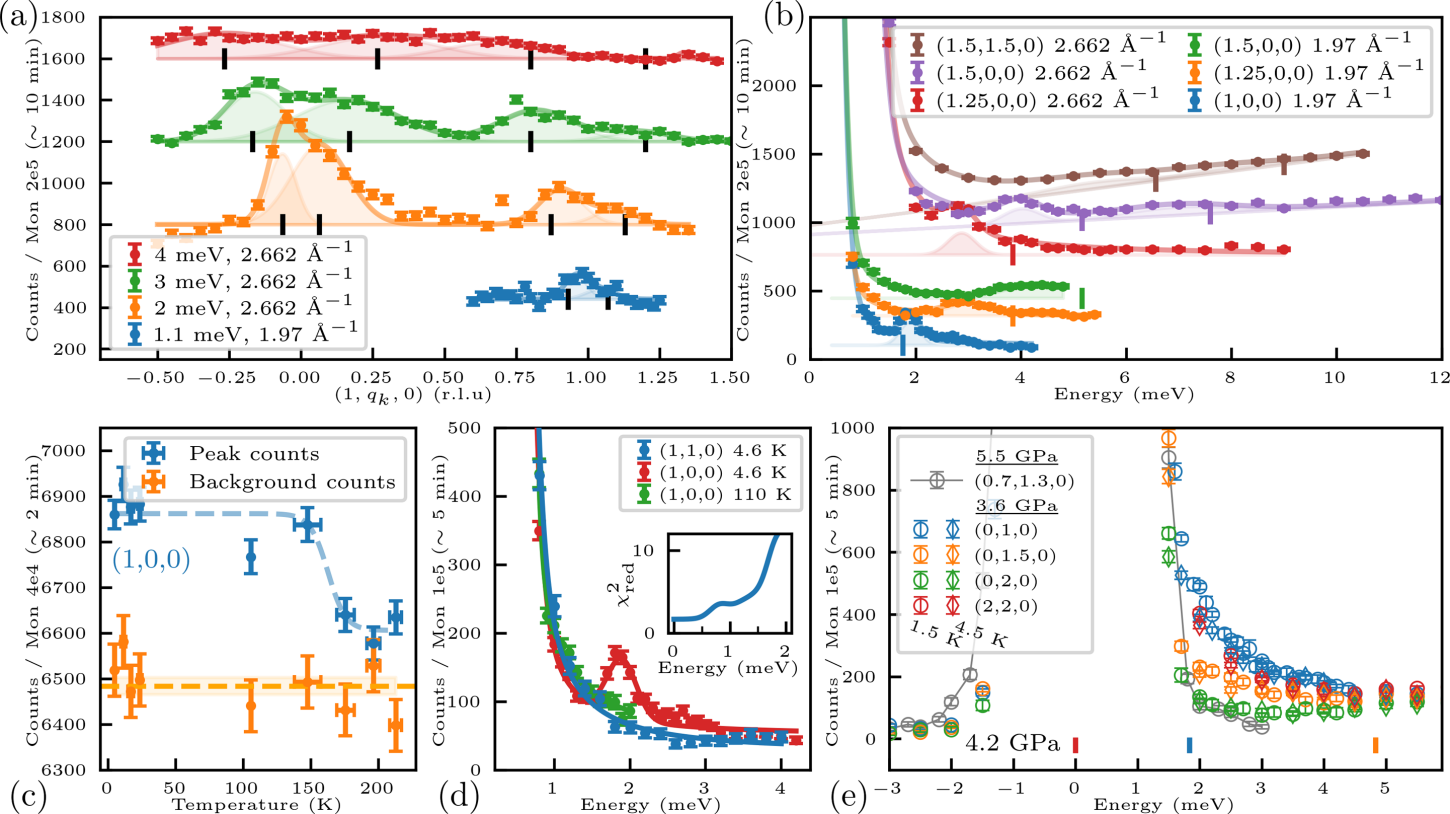
(*a*) Constant-*E* scans at (1, *q*_*k*_, 0) and (*b*) constant-*Q* scans performed with two different fixed *k*_f_. For both panels (*a*) and (*b*), the fitted mode positions are shown with the colored lines and filled-in areas as described in the text. The predicted mode positions from linear-spin-wave calculations are plotted as vertical black bars and colored using the our fitted model parameters. (*c*) The 100 magnetic Bragg peak intensity as a function of temperature at 4.2 GPa. The dashed lines are guides to the eye. (*d*) Neutron intensity as a function of energy transfer at **Q** = (1, 1, 0) and (1, 0, 0) measured at 4.6 K and approximately 110 K with *k*_f_ = 1.97 Å^−1^. The inset shows the effect on the fit quality when including an additional Gaussian peak with the same width and area as the mode observed at (1, 0, 0). The goodness of fit remains unchanged until about 0.5 meV, indicating that if a spin gap exists it must be smaller than 0.5 meV. (*e*) Constant-*Q* scans at 3.6 GPa. Expected AFM spin-wave mode positions (from the 4.2 GPa exchange parameters) are indicated by colored vertical bars for each **Q**. For **Q** = (0, 2, 0), the expected mode is at 6.1 meV. Gray points: reference scan at **Q** = (0.7, 1.3, 0).

Fig. 2 ▸(*c*) shows the temperature dependence of the 100 magnetic Bragg peak. Five sample rotation points were collected: two off-peak for background (orange) and three near the peak to approximate the integrated intensity (blue). Dashed lines are guides to the eye. Below 150 K, additional intensity appears at the (1, 0, 0) position, demonstrating the onset of AFM order. The apparent ordering temperature is slightly higher than previously reported (Haravifard *et al.*, 2014 ▸), probably due to thermal lag between the thermometers and the sample during heating.

A selection of constant-*E* scans is shown in Fig. 2 ▸(*a*). Mode positions were obtained by fitting the observed neutron intensities with Gaussian functions. These were constrained to be symmetrically displaced, in *q*_*k*_, around either (1, ±*q*_*k*_, 0) or (1, 1 ± *q*_*k*_, 0), while the widths and intensities of the peaks were left as free parameters.

A selection of constant-*Q* scans is shown in Fig. 2 ▸(*b*). The elastic line was fitted with a Voigt function, representing the instrumental resolution as a convolution of a Gaussian with a Lorentzian broadening. A sloping background was included, and the magnetic excitations were modeled with Gaussian peaks. Mode positions were then extracted from the fitted peak centers.

In Fig. 2 ▸(*d*) we compare constant-*Q* scans at the key positions (1, 0, 0) and (1, 1, 0). At base temperature a clear excitation is observed at 1.89 (2) meV at (1, 0, 0). It disappears at high temperatures, confirming its magnetic origin. This magnetic signal is not seen at (1, 1, 0). We investigate the possibility of a low-energy mode at this position by including an additional Gaussian peak with a fixed position close to the elastic line and the same width and integrated intensity as the mode observed at (1, 0, 0), since a strong mode is in general expected at the Γ point. In the inset of Fig. 2 ▸(*d*) we plot the resulting reduced chi-squared as a function of peak position with 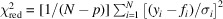. Here *N* is the number of data points, *p* the number of fitted parameters, *y*_*i*_ the measured intensities, *f*_*i*_ the model values and σ_*i*_ their respective uncertainties. The additional mode does not improve the fit quality and for mode placement above 0.5 meV the quality significantly decreases, indicating that our data do not support the presence of a gapped mode at (1, 1, 0).

Therefore, our results are consistent with the existence of a Goldstone mode at (1, 1, 0) and favor the model previously put forward including an interlayer coupling rather than the Dzyaloshinskii–Moriya interaction (Fogh *et al.*, 2024 ▸). A comparison between predicted mode positions for the two models and measured spectra shown in Figs. 1 ▸(*c*) and 1 ▸(*d*) underlines this point.

Using the implementation of linear-spin-wave theory in the *Sunny* package (Dahlbom *et al.*, 2025 ▸) for evaluating the excitation spectrum, we extracted the model parameters shown in Table 1 ▸, and these are compared with those given by Fogh *et al.* (2024 ▸). There is a good agreement between the results, keeping in mind that the two experiments were performed at different pressures. In particular, the decrease in *J* is consistent with the expected behavior of this interaction at higher pressure. This interaction is highly pressure sensitive because it goes via the Cu–O–Cu superexchange path which has a bond angle close to the Goodenough–Kanamori critical angle of 95° (Goodenough, 1955 ▸; Kanamori, 1959 ▸). On the other hand, *J*′ changes less because the superexchange path is via the stiff BO_3_ unit. The interlayer coupling also increases with pressure, as would be naively expected when increasing orbital overlaps without changing the bond angles.

Since the splitting between gapped and non-gapped modes at (1, 0, 0) is given by Δ = 8*S*(*J*′*J*_c_)^1/2^, where *S* = 1/2 is the spin quantum number of the Cu^2+^ ions (Fogh *et al.*, 2024 ▸), and the bandwidth given by *J*′ is not well determined in our experiment, the quantity that we can determine to a high precision is the product *J*′*J*_c_. Nevertheless, the results highlight the importance of the three dimensionality in the system, which may also be important at lower pressures. The interlayer coupling has little consequence in the dimer phase, but theoretical studies show that the plaquette phase in the SSM destabilizes above a critical value of *J*_c_/*J* ∼ 0.0–0.05 (Koga, 2000 ▸; Vlaar & Corboz, 2023 ▸). Although the values in Table 1 ▸ fall well below this point, the three dimensionality in SrCu_2_(BO_3_)_2_ may still play a role in the formation of plaquettes (Zayed *et al.*, 2017 ▸; Corboz & Mila, 2013 ▸; Boos *et al.*, 2019 ▸) as well as the predicted deconfined quantum critical point around the plaquette–AFM transition (Lee *et al.*, 2019 ▸; Yang *et al.*, 2022 ▸; Cui *et al.*, 2023 ▸; Guo *et al.*, 2025 ▸).

Inspecting the data collected at 3.6 GPa and shown in Fig. 2 ▸(*e*) we observe broad magnetic excitations at (2, 2, 0), (0, 1, 0) and (0, 1.5, 0), while no clear signal is observed at (0, 2, 0). When contrasted with the scattering expected for an AFM phase, using the exchange interaction obtained from the 4.2 GPa measurements, it becomes evident that the observed spectrum is not compatible with the same AFM phase. Similarly, the data are not consistent with the sharp, well defined excitations expected for the plaquette state (Zayed *et al.*, 2017 ▸). The broad spectral weight is, on the other hand, not inconsistent with the continuum-like response predicted by Corboz *et al.* (2025 ▸) and as such could be a first glimpse of the anticipated QSL phase. While the limited counting statistics and the relatively coarse energy resolution prevent any conclusive characterization of the excitation spectrum at this pressure, these preliminary results highlight the need for further experiments to elucidate this pressure region and the possible existence of a QSL in the phase diagram of SrCu_2_(BO_3_)_2_.

## 4. Conclusion

We have investigated the spin excitation spectrum of SrCu_2_(BO_3_)_2_ at 4.2 GPa using inelastic neutron scattering. Our measurements reveal dispersing magnetic excitations consistent with long-range AFM order. We use linear-spin-wave calculations to fit model parameters, obtaining values close to those previously reported at higher pressure (Fogh *et al.*, 2024 ▸). Most importantly, our measurements confirm the existence of a Goldstone mode at **Q** = (1, 1, 0) which further supports the model. In particular, we confirm that an interlayer coupling is needed to explain the measured spectrum, and hence three dimensionality may be important in SrCu_2_(BO_3_)_2_ also at lower pressures. Moreover, our findings underscore the feasibility and importance of precise inelastic neutron scattering studies under extreme conditions and provide valuable input for ongoing theoretical efforts to map the complete pressure–temperature phase diagram of SrCu_2_(BO_3_)_2_.

## Supplementary Material

Spin waves in the antiferromagnetic phase of the Shastry-Sutherland compound, SrCu2(BO3)2: https://doi.org/10.5291/ILL-DATA.4-01-1876

Magnetic excitation spectrum at the deconfined quantum critical point of the Shastry-Sutherland compound, SrCu2(BO3)2: https://doi.ill.fr/10.5291/ILL-DATA.4-01-1655

## Figures and Tables

**Table 1:** Comparison of model parameters determined in this work and given by Fogh *et al.* (2024 ▸)

	*P* (GPa)	Δ (meV)	*J* (meV)	*J*′ (meV)	*J*_c_ (meV)	*J*′/*J*	*J*_c_/*J*
This work	4.2 (2)	1.83 (6)	2.5 (2)	4.3 (1)	0.049 (6)	1.7 (2)	0.019 (2)
Fogh *et al.* (2024 ▸)	5.5 (5)	1.90 (8)	2.26 (26)	4.28 (14)	0.053 (3)	1.8 (2)	0.023 (3)

## Data Availability

Neutron data were obtained using IN8 at the Institut Laue–Langevin with support from proposal 4-01-1876 (Zayed *et al.*, 2025 ▸) and proposal 4-01-1655 (Fogh *et al.*, 2021 ▸).
